# Whole-Genome Sequencing Identified New Structural Variations in the *DMD* Gene That Cause Duchenne Muscular Dystrophy in Two Girls

**DOI:** 10.3390/ijms241713567

**Published:** 2023-09-01

**Authors:** Natalie Pluta, Arpad von Moers, Astrid Pechmann, Werner Stenzel, Hans-Hilmar Goebel, David Atlan, Beat Wolf, Indrajit Nanda, Ann-Kathrin Zaum, Simone Rost

**Affiliations:** 1 Department of Human Genetics, University of Würzburg, 97074 Würzburg, Germany; 2 Department of Pediatrics and Neuropediatrics, DRK Kliniken Berlin, 14050 Berlin, Germany; 3 Department of Neuropediatrics and Muscle Disorders, Medical Center—University of Freiburg, Faculty of Medicine, University of Freiburg, 79106 Freiburg, Germany; 4 Department of Neuropathology, Charité—Universitätsmedizin Berlin, Corporate Member of Freie Universität Berlin and Humboldt Universität zu Berlin, 10117 Berlin, Germany; 5 Phenosystems SA, 1807 Blonay, Switzerland; 6 iCoSys, University of Applied Sciences Western Switzerland, 1700 Fribourg, Switzerland; 7 Medical Genetics Center (MGZ), 80335 Munich, Germany

**Keywords:** DMD, Duchenne muscular dystrophy, whole-genome sequencing, WGS, translocation, structural variants, X-inactivation

## Abstract

Dystrophinopathies are the most common muscle diseases, especially in men. In women, on the other hand, a manifestation of Duchenne muscular dystrophy is rare due to X-chromosomal inheritance. We present two young girls with severe muscle weakness, muscular dystrophies, and creatine kinase (CK) levels exceeding 10,000 U/L. In the skeletal muscle tissues, dystrophin staining reaction showed mosaicism. The almost entirely skewed X-inactivation in both cases supported the possibility of a dystrophinopathy. Despite standard molecular diagnostics (including multiplex ligation-dependent probe amplification (MLPA) and next generation sequencing (NGS) gene panel sequencing), the genetic cause of the girls’ conditions remained unknown. However, whole-genome sequencing revealed two reciprocal translocations between their X chromosomes and chromosome 5 and chromosome 19, respectively. In both cases, the breakpoints on the X chromosomes were located directly within the *DMD* gene (in introns 54 and 7, respectively) and were responsible for the patients’ phenotypes. Additional techniques such as Sanger sequencing, conventional karyotyping and fluorescence in situ hybridization (FISH) confirmed the disruption of *DMD* gene in both patients through translocations. These findings underscore the importance of accurate clinical data combined with histopathological analysis in pinpointing the suspected underlying genetic disorder. Moreover, our study illustrates the viability of whole-genome sequencing as a time-saving and highly effective method for identifying genetic factors responsible for complex genetic constellations in Duchenne muscular dystrophy (DMD).

## 1. Introduction

Duchenne muscular dystrophy (DMD, MIM #310200) is the most frequent inherited muscle disorder caused by truncating mutations, mainly frameshift deletions or nonsense variants, in the huge *DMD* gene located on Xp21.2-p21.1 (MIM *300377). Due to the X-linked recessive inheritance, most of the affected patients are males suffering from typical symptoms as waddling gait, positive Gowers sign as a result of proximal muscle weakness, pseudohypertrophy mainly of the calf muscle, muscle weakness, cardiomyopathy, scoliosis, contractures, and/or respiratory problems [1,2].

Most female carriers usually develop no symptoms, and only 2.5 to 22% show signs such as elevated CK levels, muscle weakness, or dilated cardiomyopathy, which can be very subtle in severity [3,4,5]. In 8% of female carriers, cardiomyopathies are reported in middle or late adulthood [3].

There are various methods for diagnostic confirmation of DMD and its differentiation from other neuromuscular diseases. Examination of muscle biopsies is still a widely used method to identify the lack or reduction of dystrophin preferably with parallel re-expression of utrophin. In addition, SDS-PAGE analysis can show whether the protein in question is present or not, or whether its size has changed [6]. 

Genetic diagnostics of DMD usually starts with the detection of copy number alterations using multiplex ligation-dependent probe amplification (MLPA) to exclude exon deletions or duplications. By means of this analysis, 71–78% of patients can be properly diagnosed [7,8]. If no deletions and duplications are detected, the coding sequence of *DMD* is analysed for point mutations using Sanger sequencing or, nowadays, next generation sequencing. According to this, in about 2–7% of DMD patients, no causative variants can be detected [9,10]. One reason for these unsolved cases could be that *DMD*, as the largest gene in humans, consists of approx. 99% non-coding sequence [11], which is normally not analysed in routine genetic diagnostics. In previous studies [12,13,14,15], deep intronic variants in *DMD* have repeatedly been found to be causative for patients’ phenotypes, e.g., by inducing pseudoexons due to altered splicing or structural events such as inversions [16,17] in the large *DMD* introns. One possibility to detect splicing defects due to deep intronic variants is the analysis of mRNA extracted from the tissue in which the gene of interest is expressed. *DMD* is expressed in different tissues, such as blood, retina, peripheral nerves, and brain, but mainly in muscle tissue, and all these transcripts are tissue-specific [11]. Another option for detecting deep intronic or regulatory variants is to sequence a gene in its entirety, including all introns and 5′-/3′-untranslated regions (UTRs) [18,19], or sequencing the whole genome [20].

Here, we report on two girls in unrelated families with distinct symptoms of a muscular dystrophy and no pathogenic variants detected in the *DMD* gene or genes associated with other types of muscular dystrophies, especially limb girdle muscular dystrophies (LGMDs), within the scope of routine molecular diagnostics. Whole-genome sequencing and further analyses were applied in order to detect and to characterise the molecular cause of the muscle disease they suffer from.

## 2. Results

### 2.1. Morphological Analysis of Skeletal Muscle

**Figure 1:**
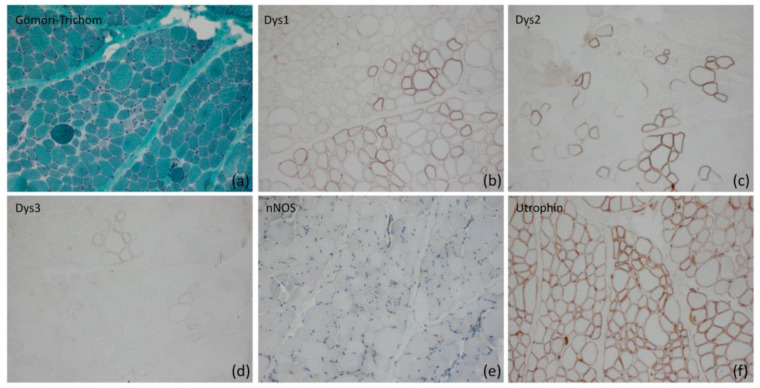
Histological analysis including immunohistochemistry of the skeletal muscle tissue of patient 1. (**a**) Atrophic and round fibers alternating with hypertrophic fibers in a Gömöri trichrome stain (original magnification ×200). (**b**) Loss of dystrophin 1 on the sarcolemma of many fibres as well as reduced staining intensity on many of them in a Dys1 (rod domain) stain (original magnification ×200). (**c**) Loss of dystrophin 2 on the sarcolemma of many fibres as well as reduced and irregular/incomplete staining intensity on many of them in a Dys2 (C-terminus) stain (original magnification ×200). (**d**) Severe loss of dystrophin 3 on the sarcolemma of many fibres approaching complete loss of staining in a Dys3 (N-terminus) stain (original magnification ×200). (**e**) Loss of nNOS on the sarcolemma of myofibres in an nNOS stain (original magnification ×200). (**f**) Compensatory upregulation of utrophin with different intensity on the sarcolemma of the myofibres in an utrophin stain (original magnification ×200).

**Figure 2:**
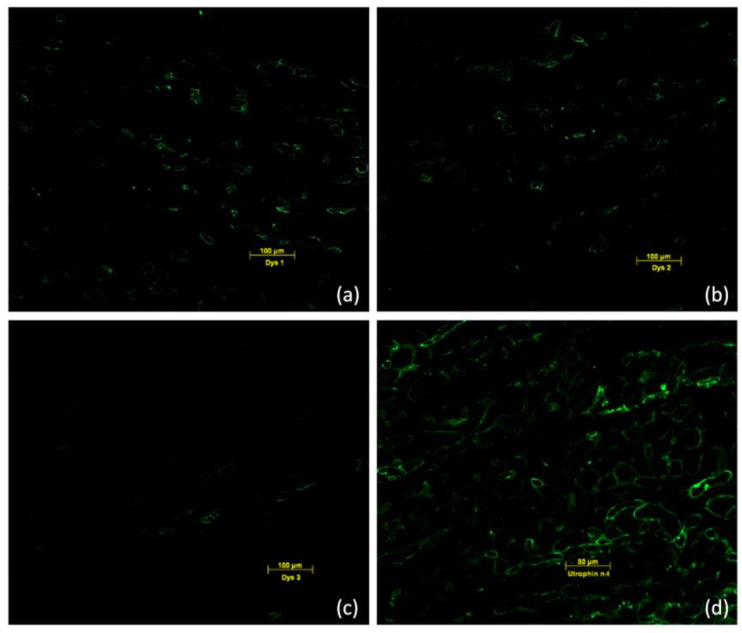
Immunofluorescence staining with antibodies against Dys1 (**a**), Dys2 (**b**), and Dys3 (**c**) showing the reduced dystrophin in most muscle fibers. Compensatory upregulation of utrophin (**d**).

The muscle biopsy (M. vastus femoris lateralis) of patient 1 showed many atrophic, predominantly round fibres alternating with hypertrophic fibres in a Gömöri trichrome stain (Figure 1a) with fibre type disproportion (predominance of small type I fibers). Immunohistochemistry by staining of dystrophin-1 (Figure 1b) and -2 (Figure 1c) revealed loss of sarcolemmal staining in most of the fibres, and staining of dystrophin-3 (Figure 1d) showed a complete loss of membrane-bound immunoreactivity, as was nNOS immunoreactivity (Figure 1e). Utrophin (Figure 1f) and laminin alpha-5 were compensatorily upregulated in the sarcolemma on all fibres. 

Immunohistology of the muscle biopsy of patient 2 showed a dystrophic picture with fibrous necrosis and connective tissue proliferation with absent staining of dystrophin with three different antibodies in most muscle fibers (Figure 2a–c) and a compensatorily upregulated utrophin (Figure 2d). All staining results pointed to a manifest dystrophinopathy in the female patients.

### 2.2. Genome Analysis

Routine diagnostic analyses by *DMD* MLPA, and muscle gene panels showed no (likely) pathogenic variants in the coding regions of *DMD* or any LGMD-associated gene (especially *SGCA*, *SGCB*, *SGCD*, *SGCG*, *ANO5*, *CAPN3*, *DYSF*, *FKRP*, *TCAP*, *CAV3*, *PYGM*, *MYOT*) in both index patients.

**Figure 3:**
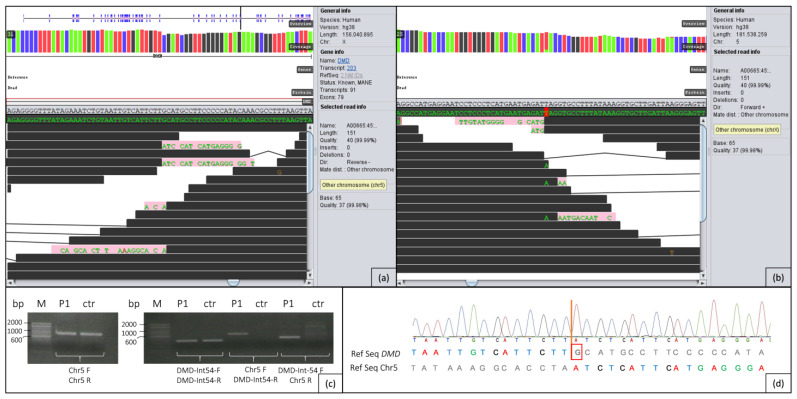
(**a**) Sequence surrounding the breakpoint (g.31628083) in intron 54 of *DMD* on chromosome X. Black lines show the sequence of the intron that matches the reference sequence; pink lines show parts of the sequence of chromosome 5 not matching the reference sequence of *DMD*. (**b**) Sequence surrounding the breakpoint (g.40072329) on chromosome 5. Black lines show the sequence of chromosome 5 that matches the reference sequence; pink lines show the sequence of intron 54 of the *DMD* gene not matching the reference sequence on chromosome 5. (**c**) PCR fragments containing the breakpoints on chromosomes 5 and X in patient 1 (P1) and a healthy control (ctr) beside a 100 bp ladder (M). The clean blank values were removed from the gel image for better clarity. (**d**) Electropherogram of the fragment amplified with primers Chr5-F and DMD-Int54-R compared to the reference sequence (Ref Seq) of *DMD* and chromosome 5. The red line indicates the breakpoint. The guanine in *DMD* outlined in red is deleted due to the translocation. On the left, patient 1 sequence matches the reference of the *DMD* gene, and on the right, patient 1 sequence matches the reference on chromosome 5.

In the whole genome data of patient 1, initial analysis of the complete *DMD* gene revealed no evident single nucleotide variants (SNVs) or conspicuous copy number variations (CNVs). Further scanning of anomalies in the whole *DMD* gene using GensearchNGS revealed a striking region in intron 54 (Figure 3a) showing reads which only partially align to this intron and additionally to a region on chromosome 5 (Figure 3b). The region on 5p13.1 (around g.40072329) does not contain any genes. It should be noted that many paired reads of this region, which are usually in close proximity to each other (in general not more than 300 bp distant), are now located on chromosome 5.

**Figure 4:**
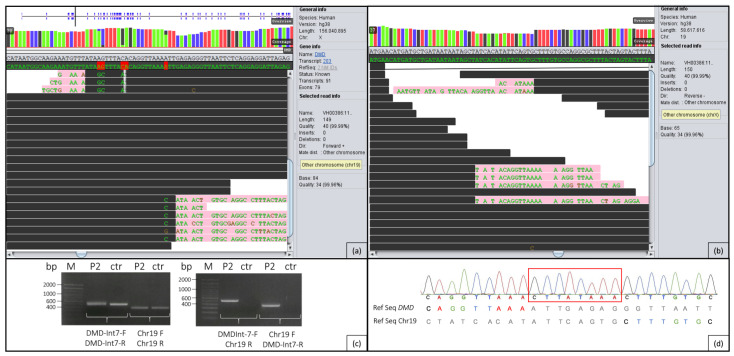
(**a**) Sequence surrounding the breakpoint area (g.32798883–g.32798891) in intron 7 of *DMD* on chromosome X. Black lines show the sequence of the intron that matches the reference sequence; pink lines show parts of the sequence of chromosome 19 not matching the reference sequence of *DMD*. (**b**) Sequence surrounding the breakpoint area (g.39820677–g.39820693) on chromosome 19. Black lines show the sequence of chromosome 19 that matches the reference sequence; pink lines show the sequence of intron 7 of the *DMD* gene not matching the reference sequence on chromosome 19. (**c**) PCR fragments containing the breakpoints on chromosomes 19 and X in patient 2 (P2) and a healthy control (ctr) beside a 100 bp ladder (M) Two additional controls and the clean blank value were removed from the gel image for better clarity. (**d**) Electropherogram of the fragment amplified with primers DMD-Int7-F and Chr19-R compared to the reference sequence (Ref Seq) of *DMD* and Chr19. The sequence outlined in red is gained due to translocation. The breakpoint lies in this area. On the left, patient 2 sequence matches the reference of the *DMD* gene, and on the right, patient 2 sequence matches the reference on chromosome 19.

In patient 2, the first step was to search for abnormalities in the entire *DMD* gene. This revealed a conspicuous region in intron 7 (Figure 4a), where parts of the reads aligned to this intron and additionally to a region on chromosome 19 (Figure 4b). No genes are known in this region on 19q13.2 (around g.39820693). While paired reads are usually no more than approx. 300 bp apart, in this case many paired reads are located on chromosome 19, in addition to the mismatching reads.

### 2.3. PCR/Sanger Analysis

The first hint for a reciprocal translocation between chromosomes X and 5 could be verified in patient 1 by PCR (Figure 3c) with subsequent Sanger sequencing (Figure 3d) of the region containing both breakpoints. The breakpoint on chromosome X is located at the genomic position g.31628083, and the breakpoint on chromosome 5 is at g.40072329 (Figure 3a,b). Figure 3c shows that in the patient, all primer combinations resulted in distinct PCR products, confirming the heterozygous translocation between chromosomes X and 5. In the control person, as expected, only fragments of the intron 54 region and of chromosome 5 are formed. Sanger sequencing clearly shows that one nucleotide (G) is lost in *DMD* due to the translocation (Figure 3d). In the PCR analysis of the parental DNA, the breakpoints in *DMD* and on chromosome 5 could not be detected.

In patient 2, PCR (Figure 4b) followed by Sanger sequencing (Figure 4c) across both breakpoints verified the initial evidence of a reciprocal translocation between chromosomes X and 19. Due to a sequence gain in the breakpoint regions of both chromosomes, the breakpoints could only be estimated. On chromosome X, the breakpoint region is located between the genomic positions g.32798883 and g.32798891, and on chromosome 19, between g.39820677 and g.39820693 (Figure 4a,b). As can be seen in Figure 4c, all primer combinations resulted in PCR products in the patient, confirming the heterozygous translocation. Figure 4c also shows that in the control person, only fragments of intron 7 of *DMD* and the intergenic region of chromosome 19 were expectedly amplified. The translocation results in an insertion of an 8 bp sequence in *DMD* (Figure 4d). PCR analysis of the parental DNA showed that both parents are not carriers of the translocation.

### 2.4. Chromosome Analysis

**Figure 5:**
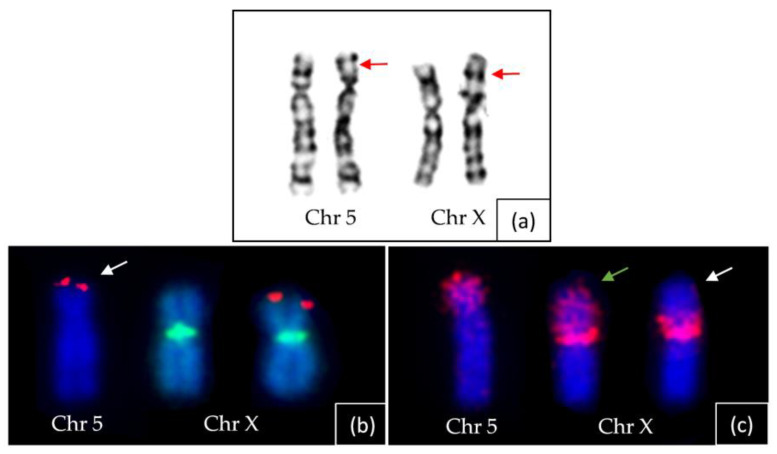
(**a**) GTG banding of chromosome 5 (Chr 5) and X (Chr X) of index patient 1. The derivative chromosomes are marked with arrows. (**b**) FISH with two different probes labeling SHOX on the terminal site of the Xp of the normal chromosome X in red and the centromere of chromosome X with DXZ1 in green. The white arrow points to the derivative chromosome 5 displaying SHOX signal at the terminal region of 5p. (**c**) FISH with probe ASP (arm-specific paint)-Xp which marks the complete short arm of the normal X chromosome in red (green arrow). The white arrow marks the derivative X chromosome showing partial labelling close to the centromere. Note near-complete labelling of the short arm of the derivative chromosome 5.

Patient 1’s chromosome analysis using conventional GTG banding did not provide sufficient resolution to diagnose the translocation, as depicted in Figure 5a. However, an unusual banding pattern suggested a likely rearrangement in the short arms of one of the homologous chromosomes 5 and X.

Additional analysis was conducted using FISH probes against specific genetic markers. The FISH analysis involved two probes: one against the SHOX gene, located at the terminal region of the short arm of chromosome X (p22.33), labelled in red; and another probe against the DXZ1 alpha satellite, specific to the chromosome X centromere, labelled in green.

The FISH results revealed a SHOX hybridisation signal at the terminal region of one of the short arms of chromosomes 5 (derivative one), while a signal was observed at the distal region of the short arm of the normal chromosome X, as shown in Figure 5b. Additionally, FISH with the TERT probe (5p15.33) displayed a signal on the terminal region of the derivative Xp, while no hybridisation signal was detected at the terminal region of the derivative 5p. Moreover, the DXZ1 hybridisation signal was present in the centromeric region of both X chromosomes.

Furthermore, the FISH analysis utilised an arm-specific paint probe (Xp), which labelled the complete short arm of one of the chromosomes 5 and simultaneously showed the absence of a hybridisation signal on most of the derivative Xp, as depicted in Figure 5c.

Consequently, the extensive FISH analyses conclusively confirmed the occurrence of a reciprocal translocation between chromosomes 5p and Xp. The breakpoints on both these chromosomes can be assigned to 5p13 and Xp22.1, respectively.

**Figure 6:**
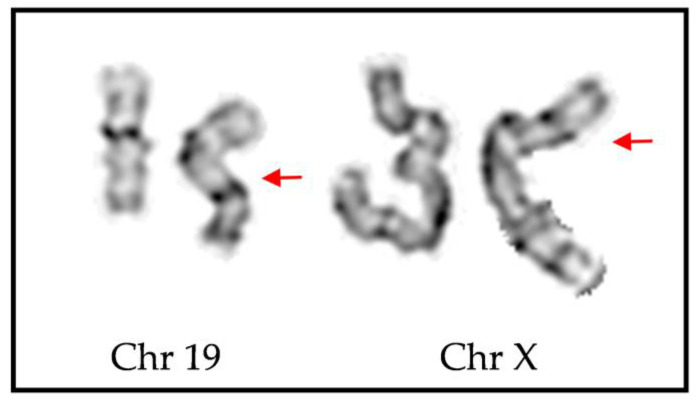
GTG banding of chromosome 19 (Chr 19) and X (Chr X) of index patient 2. The possible breakpoints involving the translocation between the long arm of chromosome 19 and the short arm of chromosome X are marked with the red arrows.

In patient 2, the translocation between chromosomes 19 and X was thoroughly examined and confirmed using conventional GTG banding. One of the homologous chromosomes exhibited an enlarged long arm, and a prominent band was observed at the distal region of 19q. However, this band did not correspond to the expected region of chromosome 19; instead, it appeared to be part of the diagnostic band located on Xp21 (Figure 6).

Based on the banding patterns observed for both derivative chromosomes 19 and X, the provisional breakpoints in the patient’s case were determined to be within the bands 19q13 and Xp21, respectively.

### 2.5. X-Inactivation Analysis

**Figure 7:**
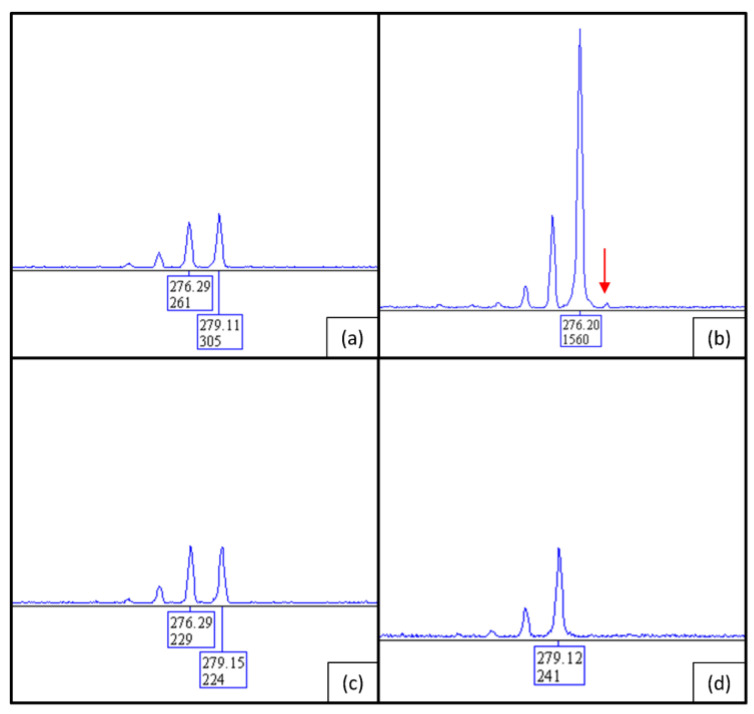
Fragment analysis of the CAG repeat in the *AR* gene of patient 1, (**a**) undigested and (**b**) digested with *HhaI*. The peak with a size of 279 bp is almost completely lost through digestion (red arrow). (**c**) Fragment lengths of the undigested AR repeats of the patient’s mother and (**d**) her father. The repeat lengths of 276 bp and 279 bp in the mother represent the two different alleles of the mother. The father has only one allele; the smaller stutter pre-peaks are artificially created by this technique.

X-inactivation analysis revealed an almost completely skewed XCI pattern in patient 1 (Figure 7a). Parallel fragment analysis of the parental DNA showed that the inactive X chromosome was inherited from the mother and the active X chromosome most likely came from the father. This could be determined by the repeat sizes of the shown fragment analysis (Figure 7): the fragment of 279 bp is present in both mother and father (Figure 7c,d), and the patient shows fragments of 279 bp and 276 bp in size (Figure 7a). Hence, the fragment of 279 bp must be inherited from the father (Figure 7d), while the fragment of 276 bp must be inherited from the mother who shows the same two alleles as her daughter (Figure 7c). By digestion with *HhaI*, which cuts unmethylated DNA, the 279 bp fragment was almost completely digested in the patient (Figure 7b). Thus, the maternal allele with a fragment size of 276 bp is still present and therefore almost completely methylated and inactive.

**Figure 8:**
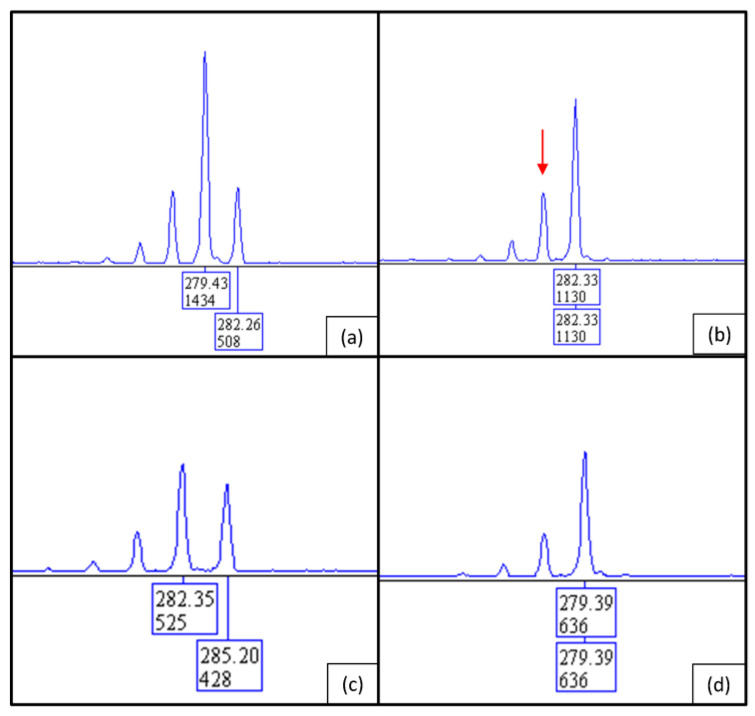
Fragment analysis of the CAG repeat in the *AR* gene of patient 2, (**a**) undigested and (**b**) digested with *HhaI*. The peak with a size of 279 bp is almost completely lost through digestion (red arrow). The peak previous to the 282 bp peak is an artifact (stutter peak of the 282 bp repeat fragment) resulting from the method used. (**c**) Fragment lengths of the undigested *AR* repeats of the patient’s mother and (**d**) her father. The repeat lengths of 282 bp and 285 bp in the mother represent the two different alleles of the mother. The father has only one allele; the smaller stutter pre-peaks are artificially created by this technique.

X-inactivation analysis of patient 2 also showed an almost completely skewed XCI pattern. Parallel fragment analysis of the parental DNA demonstrated similar results to patient 1 in that the inactive X chromosome was inherited from the mother and the active X chromosome most likely came from the father. Repeat sizes of the fragment analyses are shown in Figure 8: patient 2 has fragments of 279 bp and 282 bp in size (Figure 8a); the fragment of 282 bp was inherited from the mother (Figure 8c) and the fragment of 279 bp from the father (Figure 8d). In Figure 8b, it can be seen that the 279 bp fragment was almost completely digested by *HhaI*. Since the maternal allele of 282 bp has not been digested, it can be concluded that it is almost completely methylated and thus inactive.

## 3. Discussion

After distinct indication for Duchenne muscular dystrophy in two young girls due to detailed analysis of the muscle biopsy, whole-genome sequencing (WGS) with in-depth analysis of the *DMD* gene revealed two heterozygous reciprocal balanced translocations of chromosomes X and 5 in patient 1 as well as X and 19 in patient 2 disrupting the *DMD* gene within introns 54 or 7, respectively. The breakpoint on chromosome 5 lies in an LTR element (MSTD, RepeatMasker) [21], and the breakpoint on chromosome 19 is located in a SINE repeat element (MIRb, RepeatMasker) [21]. However, there are no repeat elements in the breakpoint regions of introns 7 and 54 in *DMD* according to RepeatMasker [21], and no homologies between the corresponding breakpoint regions were detected by BLAST (Basic Local Alignment Search Tool, National Library of Medicine, Bethesda, MD, USA). Standard diagnostics, such as MLPA, Sanger or gene panel sequencing including coding and adjacent intronic regions, could not detect these structural variants in both patients. General genome analysis by SNV and CNV detection alone was not able to clarify the molecular cause for the phenotype of patient 1. Whereas scanning the whole *DMD* gene, for structural anomalies by a special tool included in GensearchNGS (=anomaly scan) gave the first hint of a translocation between chromosomes X and 5 by partially misaligned reads in both regions. This observation could be confirmed with different molecular and cytogenetic methods. Due to the preliminary work resulting in the detection of a reciprocal translocation in patient 1, the approach concerning data analysis was different in patient 2. In this context, adaptions were made in GensearchNGS in order to detect structural variants or especially anomalies in a more efficient way. Hereupon, anomalies were scanned as a first step in patient 2, and thus, the translocation between chromosome X and 19 was detected much faster, and the diagnosis could be clarified more quickly.

The detection of (reciprocal) translocations was previously carried out with low-resolution cytogenetic methods, such as G-banding or FISH [22,23]. To further narrow down the breakpoints, specific PCRs or FISH analyses had to be carried out, e.g., using particular BAC clones as templates or probe [22,23,24].

There are several studies [25,26,27,28] in which girls with a translocation affecting the X chromosome developed DMD because the breakpoints were located in the *DMD* gene. In all the mentioned cases, the karyogram was already conspicuous in the Xp21 region where *DMD* is localised. While Nevin et al. [25] and Zatz et al. [26] postulated that *DMD* must be involved based on the localisation of the breakpoint in band Xp21 and the matching phenotype of the patients, Trippe et al. [27], additionally performed FISH analysis in order to show the breakpoint in *DMD*. In the latest paper of Szucs et al. [28], the breakpoint in *DMD* was identified with WGS. In all mentioned studies, the patients had a skewed X inactivation. To our knowledge, the identical translocations from this study have not yet been described in the literature. Although translocations between chromosome 5 and X and chromosome 19 and X with a breakpoint in *DMD* have been described before, the breakpoints in these cases were not as exactly defined as in our cases and concerned other cytogenetic bands [28,29]. 

The current study of patients 1 and 2 clearly demonstrates the limitations of conventional cytogenetic methods. In patient 1, chromosomal analysis was performed for diagnostic purposes, but a translocation was not initially considered as the cause of the muscular dystrophy.

In patient 2, the possible breakpoints on both Xp and chromosome 19q were already known when the karyogram was performed and confirmed the results based on their banding pattern and sizes (Figure 6). In chromosome 19, the enlarged long arm is due to the addition of material from Xp and cannot be accurately interpreted without suitable tools like FISH analysis or, as performed in this case, WGS

In recent studies [13,19,28,30], sequence analysis of the entire *DMD* gene was reported repeatedly as various providers offer the possibility of enriching and analysing the complete range of a gene with customised probes. In the case of *DMD*, this results in an area of approx. 2.2 MB that must be covered with specifically designed probes. While large deletions, duplications, and intronic variants can be detected with this method, it is not entirely suitable for chromosomal aberrations involving *DMD*, as it is only possible to make statements about *DMD* alone without being able to look at other chromosomes that may be involved. In addition, this method is only suitable if there is a very strong suspicion of DMD due to prior examination of a muscle biopsy. To obtain a broader view of several genes and different involved chromosomes with one single method, switching to WGS is required [19], which could also be demonstrated through both cases investigated in this study.

By means of WGS and an adequate software, it is possible to scan individual genes or defined genomic regions for anomalies which could represent structural variants. This includes areas where a part of the read does not match the reference sequence, e.g., caused by translocations. It results in a sequence that partly consists of the original sequence followed by the sequence of the translocated area. In addition, anomalies that can be detected include regions in which the paired reads are located clearly distant from each other (more than 300 bp in the cases presented here). The software used for these purposes can also distinguish whether the paired reads are within the same chromosome or located on a different chromosome. In the here presented cases, the paired reads in *DMD* intron 54 and intron 7 could be assigned to chromosome 5 and chromosome 19, respectively, which in combination with the mismatched sequences at the same location (Figure 3 and Figure 4) could suggest reciprocal translocations between chromosomes X and 5 as well as chromosomes X and 19.

In addition to the detected translocations, an almost complete skewing of X-inactivation is present in the blood lymphocytes of both girls, providing further evidence for the manifestation of the X-chromosomally, recessively inherited dystrophinopathy. The fact that DMD manifests in female carriers can have various reasons. These include mutations on both X chromosomes, either homozygous or compound heterozygous ones [31], an isodisomy of the maternal X chromosome with a pathogenic variant in *DMD* [32], Turner syndrome in patients carrying a mutation on the single X chromosome [33], women with an XY karyotype carrying a mutation in *DMD* and a (nonsense) mutation in the androgen receptor (*AR*) on the X chromosome, resulting in the female phenotype [34], or translocations between the X chromosome and an autosome with the breakpoint in *DMD* [35]. As could be shown in previous studies [36], another possibility for varying severity of symptoms or full manifestation of DMD in female carriers can be due to skewed X-inactivation. A shift can occur for several reasons, e.g., positive or negative selection depending on mutation, an older age of the patients, or chromosomal rearrangements (like translocations) [37]. It is well known that in a balanced translocation between an autosome and the X chromosome, as is the case in the here presented girls, the unaffected X becomes inactivated [38,39]. The skewed X-inactivation in the cases presented here is due to the balanced translocation between the X chromosome and chromosomes 5 or 19, and because the breakpoint lies in the *DMD* gene, the disease manifests in both female carriers.

The DNA of the parents of the girls investigated here were also analyzed by PCR, but neither of them carried the translocation in their blood. By using HUMARA assay in the patients and their parents, we were able to show that the active and thus (probably) derivative X chromosome came from the paternal germ line. This observation is not new, as there are several studies [24,40,41] in which it was shown that in the case of a translocation between an autosome and an X chromosome, the derivative X chromosome must have originated from the paternal germ line. This was the case in almost all the patients studied in these trials, and the probability that nearly every patient inherited the derivative X from their father was under 1:4000 [40,41].

In summary, WGS could detect unambiguously two structural variants, in particular translocations involving the *DMD* gene and a region without known genes on chromosome 5 or on chromosome 19 in two girls suffering from Duchenne muscular dystrophy. These cases underline the importance of WGS in combination with a complete clinical work-up of a patient including skeletal muscle biopsy for genetic diagnostics. By performing WGS as a first step, these cases could have been solved faster and with higher accuracy than by the stepwise application of conventional molecular or cytogenetic methods that were performed in routine diagnostics so far. Moreover, the standard methods of routine genetic diagnostics, especially MLPA and panel sequencing, would not be able to detect this type or similar types of structural genetic alterations. The very strong evidence of *DMD* involvement from the immunohistochemistry of the muscle biopsies made it much easier to identify the new translocations in the WGS data of the young girls.

## 4. Materials and Methods

### 4.1. Case Reports

Patient 1, a 9-year-old girl, is the second child of healthy non-consanguineous parents. She showed muscular hypotonia and delayed motor development since the first months of life. At 2 years of age, she was able to walk independently, but she had ongoing difficulties in climbing stairs and in rising up from the floor. At 4 years of age, a remarkable elevation of CK of >20,000 IU/L was detected. Standard mutation analysis of the *DMD* gene and LGMD panels revealed normal results; muscle biopsy showed the typical pattern of a dytrophinopathy. The clinical examination revealed proximal accentuated muscle weakness with positive Gowers sign, restricted walking distance, and difficulties in jumping, running, or climbing stairs. In addition to the symptoms of muscle weakness, she presented impaired cognitive skills with an IQ of 61. She is attending a school for mentally disabled children (Table 1).

After an unremarkable pregnancy and birth, patient 2 showed the first symptoms with muscular hypotonia, motor developmental delay, and failure to thrive at the age of 6 months. She gained the ability to walk independently at the age of 20 months and to climb stairs at the age of 24 months, but showed an unsteady gait pattern with frequent stumbling for a long time. The clinical examination revealed a characteristic progressive, proximally accentuated muscle weakness. The patient, who is now 14 years old, has a clearly limited ability to walk with a free walking distance of approx. 100 m. She showed normal cardiac findings and normal cognition. Repeated laboratory findings showed a significant increase in CK of 7000–12,000 U/L, already at the age of 6 months (Table 1). 

Parents as well as other family members showed no symptoms of any muscle disease. The parents of the index patients gave informed consent to all analysis performed in the context of these extended diagnostics of their daughters.

### 4.2. X-Inactivation Analysis

DNAs of the patients and their parents were isolated from whole EDTA blood samples according to standard salting-out procedures. X chromosome inactivation (XCI) was determined by HUMARA assay based on enzymatic methylation analysis of the CAG repeat in the human androgen receptor gene HUMARA (*AR*, MIM *313700) using the methylation-sensitive restriction enzyme *HhaI* and already described PCR conditions [42]. Fragment analysis was performed on an ABI 3130xl genetic analyser (Thermo Fisher Scientific, Waltham, MA, USA), and data analysis was performed using the software GeneMapper v4.0 (AppliedBiosystems, Waltham, MA, USA). 

### 4.3. DMD Analysis by MLPA and NGS

Standard diagnostic methods including multiplex ligation dependent probe amplification (SALSA MLPA Probemix P034 DMD-1 and P035 DMD-2, MRC Holland, Amsterdam, The Netherlands) for the detection of copy number variations (CNVs) in the *DMD* gene (NM_004006.2) as well as different gene panel analyses containing known OMIM genes mainly associated with muscular dystrophies were performed.

Whole genome sequencing was performed using TruSeq DNA PCR-Free Library Prep and the S1 Reagent Kit for 300 cycles on a NovaSeq 6000 sequencing system (Illumina, San Diego, CA, USA) in the case of patient 1 and the P3 Reagent Kit for 300 cycles on a NextSeq 2000 sequencing system (Illumina, San Diego, CA, USA) in the case of patient 2. Data analysis was performed after alignment of the complete genomic data against the human reference genome GRCh38 (hg38) using the software GensearchNGS v1.7.122 (PhenoSystems SA, Wallonia, Belgium) [43] with the integrated GensearchNGS aligner and internal tools for variant calling, detection of CNVs, and structural anomalies. For the detection of structural variants, the overall gene coverage was examined in detail, and CNVs as well as sequence anomalies (e.g., regions in which large parts of the sequence do not match the reference sequence, or regions in which the paired reads are located far apart from each other) were scanned and analysed in-depth using the integrated GensearchNGS anomaly scan. 

### 4.4. Sanger Sequencing and Cytogenetics

To validate the detected abnormalities within the *DMD* gene and on the affected autosomes, standard PCR comprising the potential chromosomal breakpoints followed by Sanger sequencing on an ABI 3130xl genetic analyser (Thermo Fisher Scientific, Waltham, MA, USA) were performed. Sanger data analysis was carried out by Gensearch v4.4 (PhenoSystems SA, Wallonia, Belgium). The primers shown in Table 2 were used: 

Breakpoint analysis was performed in the index patients and their parents.

Cytogenetic testing was performed using conventional karyotyping through GTG banding of metaphase chromosomes in both patients, and fluorescence in situ hybridization (FISH) was carried out in chromosomes of patient 1 with commercially available short arm-specific paint of the X (Xp) and the SHOX (Xp22.33) probes following the manufacturer’s protocol (KREATECH, Leica Biosystems Nussloch GmbH, Nußloch, Germany) A control probe DXZ1 was included in the dual color FISH experiment with the SHOX probe to identify the X chromosome. Additionally, a TERT probe from the 5p15.33 region was used to verify the transfer of 5p material to Xp.

GTG-banded metaphases at a band resolution of 450–500were used for the karyotype analysis.

## Figures and Tables

**Table 1:** Overview of the symptoms for both patients.

	Patient 1	Patient 2
age (years)	9	14
age of onset (months)	6	6
first symptoms	motor developmental delay, hypotonia	motor developmental delay, hypotonia, failure to thrive
independent walking (months)	24	20
kardiac involvement	no	no
mental involvement	yes	no
CK levels	12,000–20,000 U/L	7000–12,000 U/L
muscle biopsy	dystrophic pattern; reduction of dystrophin using dys1–3 antibodies, upregulation of utrophin	dystrophic picture with fibrous necrosis, connective tissue proliferation, absent staining of dystrophin with dys1–3 antibodies
clinical findings	proximal weakness, positive Gowers sign; walking distance, reduced; impaired cognitive skills, IQ 61	proximal weakness, walking distance 100 m;

**Table 2:** Used Primers, RefSeq Number for *DMD* NM_004006.2.

Primer Name 1	Sequence	Primer Name 2	Sequence	Fragment Size (bp)
DMD-Int54-F	5′-GGTTTGTCTCAAATTTGGCAGT-3′,	DMD-Int54-R	5′-TGGTGCACCTAGTGAACTCC-3′,	362
Chr5-F	5′-TTAGGTGGGAACATGGCATT-3′	Chr5-R	5′-TGATCTCACAGGCTCACAGC-3′	798
DMD-Int54-F	Ibid.	Chr5-R	Ibid.	472
Chr5-F	Ibid.	DMD-Int54-R	Ibid.	687
DMD-Int7-F	5′-GAGTGAATGCTTTCAGACTTGG-3′	DMD- Int7-R	5′-ATTTTCAACTGCAGAGTTTGACT-3′	488
Chr19-F	5′-GGGTTACTAATGTGTTTATTCATCTG-3′	Chr19-R	5′-CTGACCCTTTGAGCCTTGTC-3′	372
DMD-Int7-F	Ibid.	Chr19-R	Ibid.	511
Chr19-F	Ibid.	DMD- Int7-R	Ibid.	357

## Data Availability

As the data presented in this study are sequencing results of human samples, the data are not publicly available due to personal data protection.
